# Intravascular Lithotripsy for the Management of In-Stent Restenosis Due to Underexpanded Stents or Calcific Neo-Atherosclerosis: Outcomes From a Single-Center Study

**DOI:** 10.7759/cureus.79168

**Published:** 2025-02-17

**Authors:** Krishna Prasad Kurpad, Uday Kanakadandi, Naveed Adoni, Suneel Kumar, Issam D Moussa, Sanjay Mehta

**Affiliations:** 1 Cardiology, Carle Foundation Hospital, Urbana, USA; 2 Interventional Cardiology, Carle Foundation Hospital, Urbana, USA

**Keywords:** in-stent restenosis, intravascular lithotripsy, minimal stent area, neo-atherosclerosis, percutaneous coronary intervention

## Abstract

Background

Treatment of calcific neo-atherosclerosis/stent underexpansion due to inadequate calcific plaque modification continues to pose significant challenges. Intravascular lithotripsy (IVL) can cause fractures in the calcific plaques, facilitating adequate stent expansion and lumen.

Objective

Our objective is to assess the effectiveness of IVL in achieving optimal minimal stented area (MSA) in patients with in-stent restenosis (ISR) and 30-day cardiovascular and bleeding outcomes.

Methods

This is a single-center retrospective observational study conducted at a tertiary hospital. Patients who were noted to have ISR with underexpanded stents and calcific neo-atherosclerosis who did not achieve optimal MSA with non-compliant/cutting/scoring balloons were included in the study. IVL was utilized for plaque modification. Subsequently, MSA was measured post-IVL. The primary outcome included achievement of optimal MSA (defined as at least 80% of native vessel or gain in MSA of 1-2 mm²); secondary outcomes were readmission for acute coronary syndrome, major bleeding, and target lesion revascularization.

Results

A total of 19 patients and 20 lesions were included in the study. Left main (LM), left anterior descending (LAD) artery, right coronary artery (RCA), and circumflex (Cx) were culprit vessels in 5%, 60%, 10%, and 25% of cases, respectively. MSA before IVL use was 4.7, 3.23±0.41, 3.5, and 3.3±0.15, respectively, in LM, LAD, Cx, and RCA. After IVL, optimal MSA was achieved in all three vessels. The average follow-up was 3.6 months; two patients were readmitted with anemia secondary to GI bleed, while one patient was readmitted with angina and required repeat revascularization.

Conclusion

IVL is an excellent tool for the management of ISR secondary to underexpanded stents and calcific neo-atherosclerosis.

## Introduction

Intravascular lithotripsy (IVL) has grown as an excellent tool in the modification of heavily calcified plaques, thereby facilitating optimal stent delivery, adequate stent expansion, and stent opposition, which are identified risk factors for stent thrombosis [1]. The Disrupt Coronary Artery Disease (DISRUPT CAD) II trial and the DISRUPT CAD III trial have demonstrated the efficacy of IVL for the management of calcific coronary disease [1,2]. However, in-stent restenosis (ISR) presents a different challenge given the different pathophysiological processes at play. Incidence of ISR in drug-eluting stents has been reported at around 5% [3,4]. In prior studies, ISR has been defined as >50% angiographic stenosis of the stented coronary segment or within 5 mm of the prior stent edge [5]. The etiology of ISR can be divided into mechanical, biological, patient, and operator-related factors. Mechanical factors include under-expansion of the stent and stent fracture. This primarily occurs due to inadequate plaque modification of the native coronary vessel during the first implantation [6,7]. Biological factors include neo-atherosclerosis secondary to neointimal proliferation [8]. It is essential to identify the underlying mechanism using intracoronary imaging to tailor the treatment [8]. Commonly employed treatment strategies include drug-coated balloons, cutting balloons, scoring balloons, IVL, and atherectomy using Rota ablator and laser. Other treatment strategies include brachytherapy and surgical revascularization [9-13].

The underlying etiology guides treatment strategy. In patients with ISR secondary to calcific neo-atherosclerosis and underexpanded stents, IVL can be an excellent modality. Before IVL, management of these lesions would be via specialty balloons such as scoring or cutting balloons, laser, and rotational/orbital atherectomy [3]. For lesions that do not respond adequately with scoring/cutting balloons, subsequent plaque modification with laser or rotational/orbital atherectomy is necessary [3]. Even though laser and atherectomy are both efficacious, they come with inherent risks of coronary perforation and increased procedure time, among others [3]. Although there are no studies comparing modalities in a randomized fashion, prior studies on IVL have demonstrated safety [1,2]. IVL not only helps with plaque modification within the lumen, but it also creates fractures in the calcific plaque behind the stent, which leads to significant lumen gain. This has been described in case reports in the past and a few retrospective studies [14-18]. However, there is still a paucity of literature. We aim to assess the 30-day outcomes of IVL for the management of ISR secondary to underexpanded stents/calcific neo-atherosclerosis performed at our institution.

## Materials and methods

This is a retrospective observational cohort study conducted at a tertiary hospital. The study was approved by the Carle Foundation Hospital Institutional Review Board and Ethics Committee, Urbana, Illinois, US (Study Number: 23HVI3844). This research was conducted in compliance with the Helsinki Declaration 1975, as revised in 2013. This was a retrospective study, and information was obtained from medical records; hence, consent was not required for the study. The cardiac catheterization laboratory database was queried to identify all the patients who underwent percutaneous coronary intervention (PCI) for ISR with IVL (Shockwave, Shockwave Medical, Inc., USA). IVL performed for native lesions was excluded. Patients with acute coronary syndrome, including ST-segment elevation myocardial infarction (STEMI), were included in the study. Coronary angiography and intervention were performed either through the radial or femoral approach. Assessment of coronary stenosis was done through angiography. If there was significant ISR, an intravascular ultrasound (IVUS) was performed when feasible to assess the mechanism of ISR. Initial plaque modification was attempted using compliant and non-compliant balloons. Subsequently, plaque modification using either a scoring balloon (AngioSculpt, AngioScore, Inc., USA) or a cutting balloon (Wolverine, Boston Scientific Corporation, USA) was performed. If adequate reduction in stenosis is not achieved, IVL was considered, particularly if there was evidence of calcific neo-atherosclerosis or underexpanded stent due to calcific plaque behind the stent struts. If the IVL balloon did not cross the lesion, balloon dilation was performed to facilitate IVL. IVUS was performed again to confirm adequate plaque modification/expansion of the stent. Further IVUS-guided PCI with drug-eluting stent was performed at the operator's discretion.

Data was collected by the primary author, and all angiograms and IVUS images were reviewed by the senior author and were analyzed independently of the primary operator.

The primary outcome of the study was to achieve stenosis reduction of more than 80% or gain in minimal luminal area of 1-2 mm². This is based on prior studies on laser atherectomy for ISR. Furthermore, at least an MSA of 4.5 mm² was achieved.

Secondary outcomes included readmission for acute coronary syndrome, major bleeding, and target lesion revascularization at 30 days. The data supporting the findings of the article is available within the article. This study was not funded by an institution or any external grants.

## Results

A total of 19 patients (20 treated lesions) who underwent calcium modification with IVL for ISR were identified. Baseline demographics and comorbidities are presented in Table 1. The mean age of patients was 69.1±1.83 years. Thirteen (70%) patients were males. Eight (40%) cases were elective PCI for symptomatic stable CAD, while 11 (60%) patients presented with acute coronary syndrome. One patient presented with STEMI. Eleven (55%) patients had an ejection fraction <50%. Seven (35%) patients had chronic kidney disease (CKD) at baseline. With respect to baseline antiplatelet therapy, 18 (90%) patients were on aspirin 81 mg PO QD, and 11 (55%) patients were on P2Y12 inhibitors. The mean time since the last PCI was 109 months. Other salient factors have been presented in Table 1.

**Table 1:** Baseline demographics and comorbidities (n=19; 20 lesions). BMI: body mass index; CABG: coronary artery bypass grafting; PCI: percutaneous coronary intervention; ACE inhibitors: angiotensin-converting enzyme inhibitors; ARBs: angiotensin II receptor blockers; ARNIs: angiotensin receptor-neprilysin inhibitors; MRA: mineralocorticoid receptor antagonists; EF: ejection fraction

Category	Values
Demographics	
Mean age (years)	69.1±1.83
Females (%)	30
Males (%)	70
Median body mass index (BMI kg/m^2^)	30.61
Comorbidities	
Hypertension	18 (95%)
Hyperlipidemia	19 (100%)
Diabetes	10 (50%)
History of CABG	2 (20%)
Abnormal nuclear stress test	6 (30%)
Elective PCI	8 (40%)
Acute coronary syndrome	12 (60%)
Chronic kidney disease	7 (35%)
Heart failure with reduced ejection fraction (<35%)	7 (35%)
Mean time since last PCI	109 months
Baseline medications (%)	
Beta-blockers	16 (80%)
ACE inhibitors/ARBs	15 (75%)
Statins	19 (95%)
ARNIs	2 (10%)
Aspirin	18 (90%)
P2Y12 Inhibitors	11 (55%)
MRA	2 (10%)
Echocardiogram (%)	
EF<35%	5 (25%)
EF 36-50%	6 (30%)
EF>50%	8 (45%)

About procedural characteristics (Table 2), a total of 20 interventions in 19 patients were included in the study. The left main (LM) intervention was performed in one patient; the left anterior descending (LAD) and right coronary artery (RCA) represented the majority of the interventions. IVUS was used in 18 (85%) cases. All lesions had underexpanded stents due to calcific plaque behind the stent struts, while 17 (85%) out of 20 lesions also had calcific neo-atherosclerosis. The mean length was 23.75±2.33 mm. Non-compliant balloons were used as the first strategy in all the cases. Subsequently, cutting balloons and scoring balloons were used in 10 (50%) and 4 (20%) cases, respectively. Pre-lithotripsy minimal stented area (MSA in mm²) was 4.7, 3.41±0.25, 3.5, and 3.7±0.35, three in LM, LAD, circumflex (Cx), RCA, and ramus, respectively. After lithotripsy, MSA was 8.2, 6.93±0.55, 6.5, 8.78±1.03, and 5.5 in the LM, LAD, Cx, RCA, and ramus, respectively. Total impulses delivered with shockwave balloons ranged from 40 to 80 impulses. Post-lithotripsy, stenting was done in 12 (58%) cases. The mean post-procedure follow-up was 3.75 months.

**Table 2:** Procedural characteristics and outcomes (20 lesions). LAD: left anterior descending; RCA: right coronary artery; ISR: in-stent restenosis; IVUS: intravascular ultrasound; MSA: minimum stenosis area; PTCA: percutaneous transluminal coronary angioplasty; ACS: acute coronary syndrome

Features	Values
Angiogram with significant coronary stenosis n (%)	
Left main	1 (5%)
LAD	13 (65%)
Diagonal/ramus	6 (30%)
Circumflex	5 (25%)
Obtuse marginal	5 (25%)
RCA	11 (55%)
Culprit lesion (%)	
Left main	1 (5%)
LAD	11 (55%)
Circumflex	2 (10%)
RCA	4 (25%)
Ramus	1 (5%)
Lesion length (mm)±SD	23.75±2.33
Non-compliant balloon	20 (100%)
Cutting balloon angioplasty	10 (50%)
Scoring balloon	4( 20%)
Laser	1 (5%)
IVUS use	18 (85%)
Mechanism of ISR (n)	
Intimal hyperplasia	1 (5%)
Calcific neo-atherosclerosis	17 (85%)
Underexpanded stent	20 (100%)
Calcific atherosclerosis behind the stent struts	9 (45%)
Pre-lithotripsy MSA (mm^2^)	
Left main	4.7
LAD	3.41±0.25
Circumflex	3.5
RCA	3.7±0.35
Ramus	3
Post lithotripsy MSA (mm^2^)	
Left main	8.2
LAD	6.93±0.55
Circumflex	6.5
RCA	8.78±1.03
Ramus	5.5
Only PTCA	42.85%
Post-procedure mean follow up	3.75 months
Re-hospitalization: (total number)	
Heart failure	1 (5%)
ACS	1 (5%)
Anemia	2 (10%)
Target vessel revascularization	1 (5%)
Post-procedure bleeding	1 (5%)
Pseudoaneurysm	0
Anemia	2 (10%)
Acute kidney injury	0
In-hospital mortality	0

**Figure 1:**
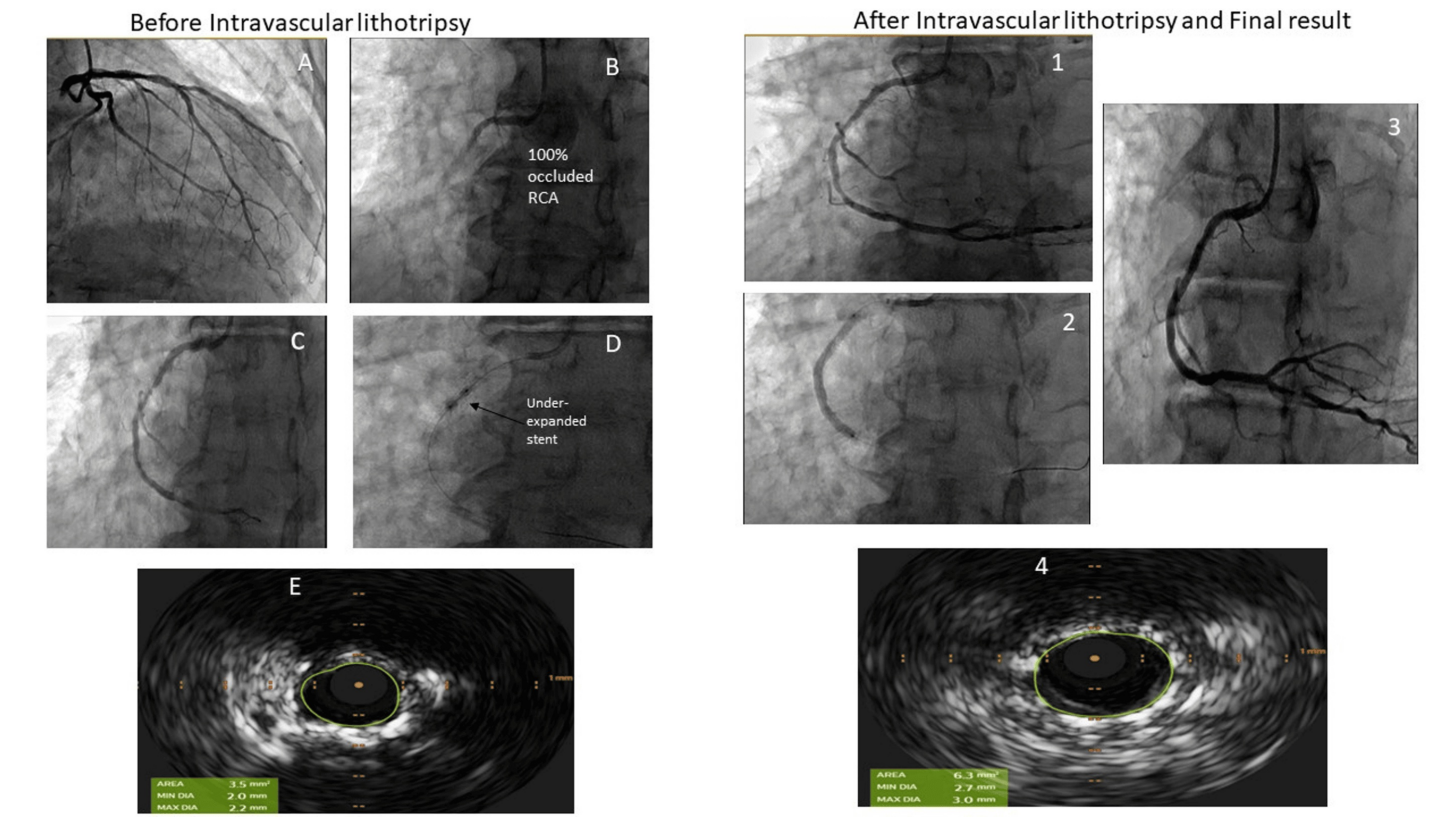
Angiogram and intravascular ultrasound pre- and post-intervention of a representative patient included in the study. Before intravascular lithotripsy A: Angiogram of the left coronary system demonstrating moderate disease in the mid-left anterior descending artery. B: Angiogram of the RCA demonstrating 100% acute occlusion in the proximal segment with TIMI 0 flow. C: RCA angiogram demonstrating 90% in-stent restenosis in the mid-segment. D: Balloon angioplasty of mid-RCA stenosis demonstrating underexpansion of the balloon. E: Intravascular ultrasound of mid-RCA lesion demonstrating severely reduced minimal stent area 3.5 mm². After intravascular lithotripsy 1: Angiogram of RCA demonstrating good expansion of lesion in the mid-RCA. 2. Deployment of the stent in the proximal and mid-RCA with good expansion 3. Final angiogram of RCA demonstrating well-expanded stent with TIMI 3 flow. 4. Intravascular imaging of RCA demonstrating good stent expansion and apposition with an increase in minimal stent area to 6.3 mm². RCA: right coronary artery

One patient presented with recurrent ISR and was a persistent heavy smoker. Other outcomes are presented in the table. The representative case has been presented in Figure 1.

## Discussion

Our study demonstrates the utility and safety of IVL for the management of ISR in patients with calcific neo-atherosclerosis and underexpanded stents. IVL has not been studied in large populations for the management of ISR secondary to underexpanded stents or calcific neo-atherosclerosis. The approach to the management of ISR is multifaceted and needs a tailored approach depending upon the mechanism at play. Mechanisms of ISR can be broadly divided into stent-related factors such as underexpansion, stent fracture, or the natural progression of disease including neointimal hyperplasia (NIH) and neo-atherosclerosis with and without calcification [8]. Stent underexpansion is often due to inadequate plaque modification, particularly in circumferential calcified lesions [6,7]. Often, mechanisms are mixed and typically require a multipronged approach for optimal results. Intravascular imaging is a key component in defining the mechanism of ISR, which further guides the treatment strategy.

In patients with calcific neo-atherosclerosis, underexpanded stents with a calcific plaque behind the stent struts and multilayer ISR (>2 stents), often optimal MSA is not achieved with a non-compliant balloon, cutting balloon angioplasty, and scoring balloon angioplasty. Atherectomy with rotational or orbital system, laser is useful in patients with calcific neo-atherosclerosis [11-13]. However, in patients with underexpanded stents with calcific plaque behind the stent struts, the options are limited. IVL, however, can modify the plaque behind the stent struts and lead to good lumen gain and optimal MSA. Sonoda et al. [19] in a randomized controlled trial demonstrated improved outcomes by achieving optimal minimal stent area (mean MSA was >5 mm² for drug-eluting stent and 6 mm² for bare metal stent).

In this retrospective study of 19 patients with 20 ISR lesions, 17 (85%) lesions were calcific neo-atherosclerotic, while nine (45%) lesions, in addition, had calcific plaque behind the stent struts. MSA, as mentioned in the table above, was sub-optimal after non-compliant balloon, cutting balloon angioplasty, and scoring balloon angioplasty. With IVL, we were able to achieve adequate MSA in all the patients. Twelve (60%) patients received an additional layer of drug-eluting stent due to the extension of plaque beyond the stent area. There was no immediate intra- or peri-procedural complication noted. One patient developed retroperitoneal bleeding requiring a blood transfusion. Two patients were readmitted to the hospital with major gastrointestinal bleeding requiring a blood transfusion. One patient, who was a persistent smoker, was readmitted with non-ST elevation myocardial infarction (NSTEMI) with a recurrence of ISR in a few months requiring intervention.

The role of IVL in underexpanded stents has been described by Pham et al. [20] in a retrospective study of 17 lesions due to underexpansion and demonstrated excellent outcomes like our study. Mechanistically, ultrasound energy modifies the calcific plaque behind the stent, allowing for adequate stent expansion. In our study, we included two patients who had acute stent underexpansion, which was detected during the procedure, and subsequently IVL was used to further modify the calcific plaque behind the stent. The effect of IVL on a newly placed drug-eluting stent, in the long run, is unknown and has not been reported before. The SMILE registry by Ielasi et al. [21] in 2020 described the use of IVL for the management of stent underexpansion with good efficacy; this study also included two patients with an acute underexpanded stent. The IVL-DRAGON registry by Wańha et al. [22] and the CRUNCH registry by Tovar Forero et al. [23], both in Europe, have reported the utility of IVL for underexpanded stents. Overall, IVL can be a bailout strategy when there is underexpansion of newly placed stents with inadequately modified calcific plaque.

Overall, this is a small retrospective observational study, and larger studies are necessary to validate the outcomes.

## Conclusions

In conclusion, IVL could be considered for the management of neo-calcific atherosclerosis and underexpanded stents both in chronic settings with calcific plaque behind the stent struts and acutely underexpanded stents. IVL has changed the paradigm of the management of calcified plaques in both native calcific CAD and calcific ISR and will be a useful tool in the future.
